# RETRACTION: The Impact of Early Nutritional Support on Postoperative Wound Healing in Patients With Complex Fractures: A Meta‐Analysis Review

**DOI:** 10.1111/iwj.70624

**Published:** 2025-04-21

## Abstract

**RETRACTION:** BaoL. , ChuR. , ZhangL. , LiJ. , YangH. , and PangH. , “The Impact of Early Nutritional Support on Postoperative Wound Healing in Patients With Complex Fractures: A Meta‐Analysis Review,” International Wound Journal 21, no. 3 (2024): e14782, 10.1111/iwj.14782.38468366 PMC10928242

The above article, published online on 11 March 2024, in Wiley Online Library (http://onlinelibrary.wiley.com/), has been retracted by agreement between the journal Editor in Chief, Professor Keith Harding; and John Wiley & Sons Ltd. Following an investigation by the publisher, all parties have concluded that this article was accepted solely on the basis of a compromised peer review process. The editors have therefore decided to retract the article. The authors did not respond to our notice regarding the retraction.

**RETRACTION:** L. Bao , R. Chu , L. Zhang , J. Li , H. Yang , and H. Pang , “The Impact of Early Nutritional Support on Postoperative Wound Healing in Patients With Complex Fractures: A Meta‐Analysis Review,” International Wound Journal 21, no. 3 (2024): e14782, 10.1111/iwj.14782.38468366 PMC10928242

The above article, published online on 11 March 2024, in Wiley Online Library (http://onlinelibrary.wiley.com/), has been retracted by agreement between the journal Editor in Chief, Professor Keith Harding; and John Wiley & Sons Ltd. Following an investigation by the publisher, all parties have concluded that this article was accepted solely on the basis of a compromised peer review process. The editors have therefore decided to retract the article. The authors did not respond to our notice regarding the retraction.

